# In-air hearing of the great cormorant (*Phalacrocorax carbo*)

**DOI:** 10.1242/bio.023879

**Published:** 2017-03-13

**Authors:** Alyssa Maxwell, Kirstin Anderson Hansen, Sara Torres Ortiz, Ole Næsbye Larsen, Ursula Siebert, Magnus Wahlberg

**Affiliations:** 1 Marine Biological Research Centre, Department of Biology, University of Southern Denmark, Hindsholmvej 11, Kerteminde DK-5300, Denmark; 2 Institute for Terrestrial and Aquatic Wildlife Research (ITAW), University of Veterinary Medicine Hannover, Foundation, Werftstrasse 6, Büsum D-25761, Germany

**Keywords:** Aquatic birds, Hearing, Psychophysics, Signal detection theory, Unbiased hearing sensitivity

## Abstract

Many aquatic birds use sounds extensively for in-air communication. Regardless of this, we know very little about their hearing abilities. The in-air audiogram of a male adult great cormorant (*Phalacrocorax carbo*) was determined using psychophysical methods (method of constants). Hearing thresholds were derived using pure tones of five different frequencies. The lowest threshold was at 2 kHz: 18 dB re 20 µPa rms. Thresholds derived using signal detection theory were within 2 dB of the ones derived using classical psychophysics. The great cormorant is more sensitive to in-air sounds than previously believed and its hearing abilities are comparable to several other species of birds of similar size. This knowledge is important for our understanding of the hearing abilities of other species of sea birds. It can also be used to develop cormorant deterrent devices for fisheries, as well as to assess the impact of increasing in-air anthropogenic noise levels on cormorants and other aquatic birds.

## Abstract (precis)

**Summary:** The in-air audiogram of a male adult great cormorant (*Phalacrocorax carbo*) was determined using psychophysics methods to further our knowledge of hearing sensitivity in aquatic birds in relation to human generated noise.

## INTRODUCTION

More than 800 out of the 10,000 identified species of birds spend a significant part of their lives in, on or close to water (Dooling and Therrien, 2012). Many aquatic birds dive for food, such as macro- or planktonic algae, fish, squid or other animals. At their resting and mating sites they are often extremely social, and use not only visual displays but also sounds to communicate (Laidre, 2012; Martin, 1998). Birds are also known to use hearing to orientate themselves, and probably to detect predators and prey (Tyack, 1998). Therefore, sound plays a vital role not only for terrestrial but also for aquatic birds.

The importance of sound is evident from the well-developed hearing abilities in most birds. Smaller birds have a hearing sensitivity range from 0.25 up to 8 kHz, whereas the frequency range as well as hearing sensitivity of larger birds is lower (except for hearing specialists, such as owls; Dooling, 1992, 2002; Dooling et al., 2000). Hearing thresholds can be measured using physiological techniques by recording from the auditory nerve or the central auditory neurons (Konishi, 1969), or by detecting the so-called auditory brainstem response (ABR; Hall, 2007). Hearing thresholds can also be determined through psychophysical testing, where the animal is asked whether it hears a signal or not (Fay, 1988; Chapter 3 in Gescheider, 1997). Physiological measurements usually produce data more rapidly than psychophysical experimentation. However, thresholds derived using, for example, ABR have not considered the full auditory pathway from the outer ear to the cortex of the brain, as is the case for psychophysical thresholds. It is therefore important to calibrate physiologically derived thresholds with psychophysical experimentation.

Present data on the in-air hearing abilities of aquatic birds has been obtained using both ABR (Crowell et al., 2015) and psychophysical (Johansen et al., 2016) techniques. In these studies, the hearing thresholds varied by more than 20 dB for a certain frequency, both between different species (Crowell et al., 2015) but also between research sessions in a study of a single individual (Johansen et al., 2016). It is presently not clear if these large variations were caused by methodological issues or actual variations in the birds' hearing sensitivity. The ABR recordings were obtained from anaesthetized birds and may have been affected by, for example, the placement of the electrodes or by the anaesthesia (Crowell et al., 2015). The psychophysical data was collected in open air (Johansen et al., 2016), and even though there were no indications of the hearing thresholds being masked by background noise, various noise sources may still have affected the attention of the bird during these trials. Therefore, since it has not been possible so far to evaluate the hearing abilities of marine birds in-air with any great precision, it is of great value to obtain more precise hearing data.

A feasible model species for measuring the hearing of marine birds is the great cormorant (*Phalacrocorax carbo*). It is one of the most efficient of marine predators, foraging daily on several hundred grams of fish caught during long dives (Grémillet et al., 1998). Great cormorants adapt well to life in captivity and are known for being easy to train (Jackson, 2010). Therefore, this species is an appropriate subject for psychophysical experiments.

Hearing thresholds can be derived by measuring the so-called psychometric function, or the HIT rate (which is the fraction of correct responses when a signal is presented in the trials) as a function of stimulus levels. There is a problem, however, with this so-called classical psychophysical method (*sensu* Chapter 2 in Gescheider, 1997) as it usually does not take the animal's decision criteria into account (see, however, May et al., 1995). The way the animal responds to the stimulus may change depending, for example, on the amount or quality of the reinforcement it receives during the trials for a correct or incorrect response (Chapter 5 in Gescheider, 1997; Chapter 5 in Green and Swets, 1966).

One way to obtain a criteria-independent (so-called unbiased) measure of the animal's hearing is to use signal detection theory (SDT; Chapter 5 in Green and Swets, 1966). Here the animal's decision criterion is taken into account by combining data on both the HIT rate and the false alarm rate (FA; the rate at which the subject responds yes when there is no stimulus present). Even though signal detection theory is often used in human psychophysics to obtain unbiased measures of hearing sensitivity (see Chapter 5 in Green and Swets, 1966), it is not always implemented in psychophysical work on other species (see, however, Dooling and Okanoya, 1995; Klink and Klump, 2004; Langemann and Klump, 2001; Schusterman, 1974).

Here we estimate the audiogram of the great cormorant by conducting the study inside a soundproof chamber, which allowed for the experiments to be performed with very little disturbance from the outside environment. Thresholds obtained are comparable to the ones measured previously with similar-sized terrestrial birds (e.g. Dooling, 2002). By comparing results derived by classical psychophysical and signal detection theory techniques, we show that the measured hearing sensitivities seem robust and indicate only moderate changes in the animal's decision criteria throughout the experiment.

## RESULTS

**Fig. 1.**
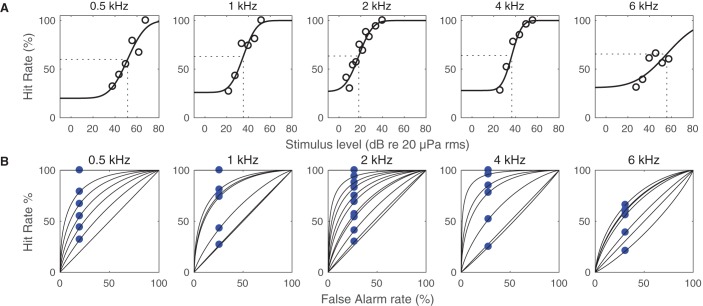
**Psychomentric functions of in-air hearing in the great cormorant.** (A) Probit curves fitted to the HIT rate as a function of stimulus level for each tested frequency. The hearing threshold from half-way between the FA rate and 100% (ranging from a HIT rate of 60 to 66%) on the probit curve is indicated. (B) ROC of in-air hearing in the great cormorant. The actual measured hit rate and false alarm rate is indicated by blue circles for each sound level. The diagonal line is the chance line.

Out of the fifty sessions performed, five were not included in the data analysis due to inappropriately high FA rates (beyond 35%). The FA rate of the used sessions were 20-31% when all catch trials for each frequency were pooled (Table 1). The psychometric functions (Fig. 1A) had R^2^ values of 0.58-0.98. The classical psychophysical hearing thresholds are listed in Table 1. These thresholds, defined as the stimulus level generating a HIT rate of the psychometric function half-way between the FA rate and 100% (ranging from a HIT rate of 60 to 66%), are shown in Fig. 1A.

**Table 1.**
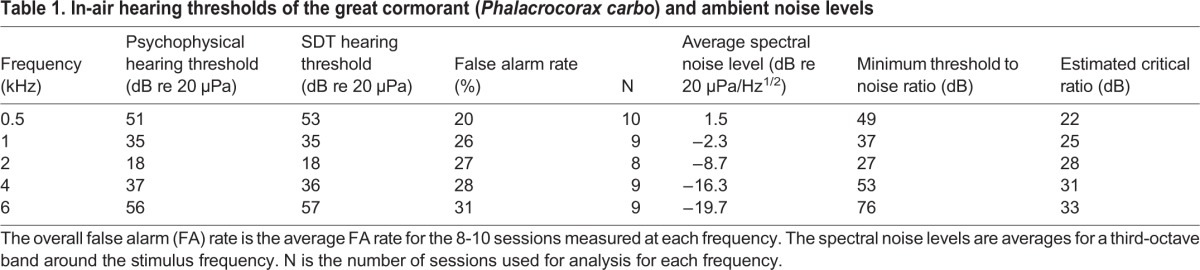
**In-air hearing thresholds of the great cormorant (*Phalacrocorax carbo*) and ambient noise levels**

ROC plots are shown in Fig. 1B for each stimulus frequency and level. The stimulus level belonging to each ROC curve are usually monotonously decreasing towards the diagonal (‘chance’) line, except for a few cases. All ROC curves are above the chance line (the positive diagonal, where the HIT rate=FA rate, so that the animal's response is identical during signal and catch trials), except for the lowest signal levels at 4 and 6 kHz, which are right below the chance line (Fig. 1B).

**Fig. 2.**
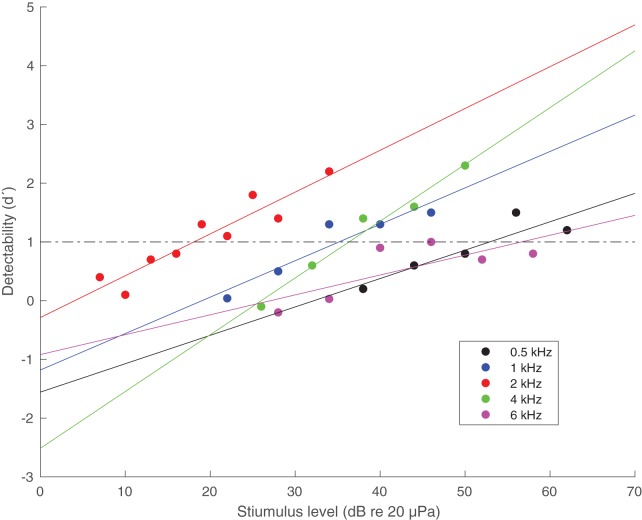
**The detectability (d’) as a function of stimulus level in psychophysical trials of the in-air hearing abilities of the great cormorant.** Coloured lines are linear regression lines used to determine an unbiased threshold of the bird's performance at d’=1 for each frequency (indicated by a dashed-dotted horizontal line).

**Fig. 3.**
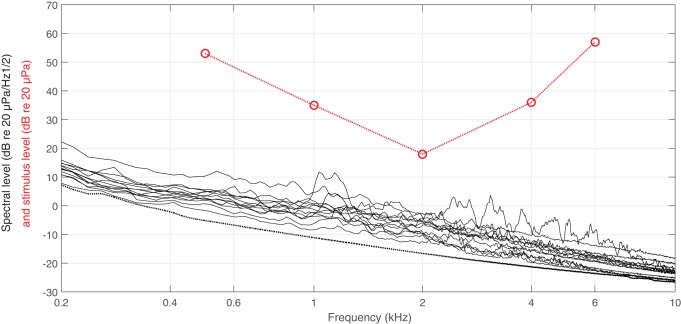
**In-air psychoacoustics audiogram for the great cormorant.** The hearing thresholds (red circles) are derived from signal detection theory, and the red stipled line connects the data points with straight lines. The ambient noise levels (black lines) were collected right before or after each research session. The dotted black line is the self-noise of the recording system. The y-axis is given simultaneously in units belonging to the hearing thresholds (in red) and to the noise measurements (in black).

In Fig. 2 the detectability (d’) is plotted as a function of stimulus level and frequency. The stimulus level giving d’ values of 1 from the linearly fitted lines were used as the hearing threshold derived by signal detection theory and is plotted as red circles in Fig. 3. The thresholds derived by classical psychophysics and signal detection theory are within 2 dB at each frequency (Table 1).

Ambient noise levels decreased with increasing frequency (Fig. 3). When measured in a third-octave band around each stimulus frequency, they were 27-76 dB below the hearing threshold at each frequency (Table 1). When compared with the estimated critical ratio (see Okanoya and Dooling, 1987) for each of the six frequencies, the threshold-to-noise ratio was always larger than the critical ratio, except for at 2 kHz where they were comparable (Table 1). At 2 kHz there is a slight risk that the hearing threshold would be masked by ambient noise in some trials. This possible issue with the data was investigated further by deriving hearing thresholds for each session (by the same probit analysis used for the cumulative data set) and comparing them with the individual third-octave band noise centred at 2 kHz, measured right before or after each session. A linear regression performed on these thresholds (varying with 18 dB between the sessions) as a function of the background noise levels (varying with 10 dB between the sessions) did not have a slope significantly different from 0 (ANOVA, *F*_1,6_=1.2, *P*=0.4). If the threshold would have been masked, we would have expected the regression line to have a significant positive slope.

No noticeable difference in neither the HIT rate (ANOVA, *F*_1,195_=0.30, *P*=0.58) nor the FA rate (ANOVA, *F*_1,194_=2.2, *P*=0.14) was seen when the double-blind sessions were compared to the non-double blind sessions (FA rate was 25% for double blind and 30% for non-double blind). Also, the FA rate showed no effect on HIT rate for each individual session (ANOVA, *F*_1,195_=0.07, *P*=0.80). There was a significant increase in HIT rate with increasing stimulus level (ANOVA, *F*_1,195_=17, *P*<0.0001), while the frequency had no effect on HIT rate (ANOVA, *F*_1,195_=0.51, *P*=0.48).

## DISCUSSION

The aim of this study was to determine the in-air hearing sensitivity of the great cormorant using behavioural testing. The results show that the cormorant audiogram follows the same U shape found in other avian audiograms (Fay, 1988). The close fit between HIT rate data and psychometric functions (Fig. 1A) indicates that the derived thresholds are probably less prone to noise than the previous data from Johansen et al. (2016) from the same individual. This is most likely due to the fact that in the present study, the bird was tested in a soundproof acoustic chamber, which allowed for fewer disturbances from the surrounding environment. Also, Johansen et al. (2016) used the method of limits (Levitt, 1971; Chapter 3 in Gescheider, 1997) rather than the method of constant stimuli (Levitt, 1971; Chapter 3 in Gescheider, 1997) used here. This may further explain some of the differences between the previously reported results by Johansen et al. (2016) and the results presented here.

The difference between the threshold and the ambient noise level was much larger than the estimated critical ratio for all measured frequencies, except at 2 kHz where they were comparable (Table 1). The fact that the 2 kHz thresholds did not vary as a function of the ambient noise level show that the stimuli were not masked. It should be remembered that the critical ratio is defined as the difference between a masked threshold and the spectral noise level for white noise, i.e. noise with no temporal structure and a flat frequency spectrum (Moore, 2012). The ambient noise present during the measurements made here does not possess any of the features found in white noise; it was both temporally and frequency modulated. When signals are detected in such noise, the detection threshold is usually much lower than what is predicted from the critical ratio (Moore, 2012). This may explain why there were no signs of masking at 2 kHz, even though the difference between the threshold and the ambient noise spectral density was very close to the estimated critical ratio. All in all, we can safely conclude that there is no risk of the background noise having masked any of the hearing thresholds presented in this study.

For 27 out of the 34 ROC curves, the curves decrease monotonically towards the chance line (Fig. 1B). This is what would be expected for a bird keeping a constant criterion throughtout the sessions. The remaining and slightly aberrant seven curves could indicate momentary variations in the animal's hearing threshold or attention during the trials. 32 out of 34 ROC curves are above the chance line, as expected for an animal properly trying to solve the psychophysical task. The two ROC curves slightly below the chance line in the 4 and 6 kHz data are probably caused by random errors.

**Fig. 4.**
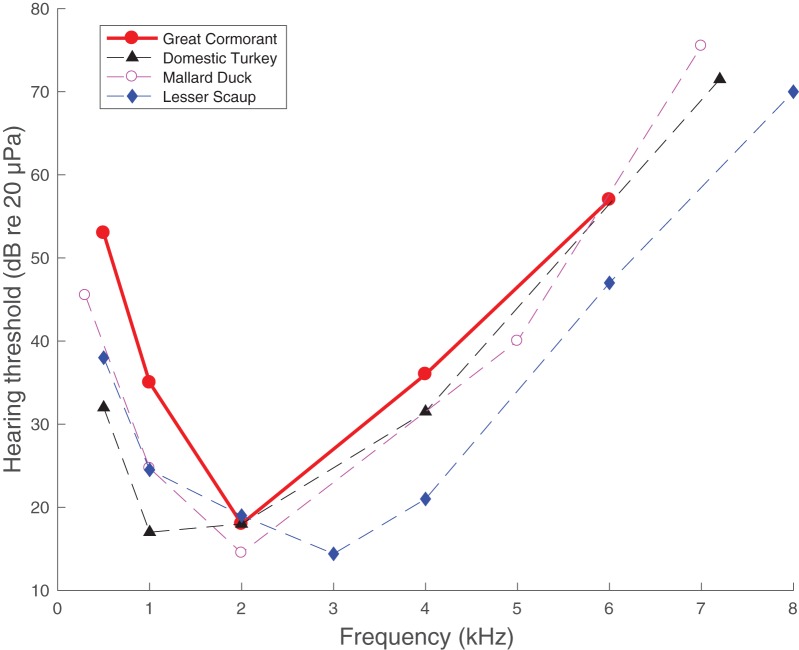
**The hearing abilities of large birds.** Comparison of hearing sensitivities of the great cormorant (*Phaloacrocorax carbo*; red: this study), a domestic turkey (*Meleagris gallopavo*; black: classical psychophysics), a mallard duck (*Anas platyrhynchos*; lilac: classical psychophysics), and a lesser scaup (*Aythya affinis*; blue: auditory brainstem response). Psychophysics data for the domestic turkey from Maiorana and Schleidt (1972), mallard duck from Trainer (1946), and lesser scaup data from Crowell et al. (2015).

The thresholds resulted in an audiogram similar in shape to that of other bird species of similar size (Fig. 4). At frequencies of 2 kHz and higher the cormorant hearing thresholds were within a few dB from the other species, but at lower frequencies they were up to 15 dB higher (Fig. 4). These threshold variations may be species-specific but could also be caused by methodological differences between the different studies. The fact that the FA rate was similar across frequencies in our study (Table 1) makes it unlikely that there would be frequency-specific biases in the threshold estimations during data collection from the cormorant.

The signal detection theory thresholds are very similar, within 2 dB, to the ones derived by classical psychophysical techniques (Table 1). Great caution is always needed when comparing the psychophysical and signal detection theory approaches for deriving hearing thresholds, not only in this study but also in other studies. The fact that the thresholds are so similar in this study indicates that the bird kept a stable criterion for detecting signals throughout the trials (see Chapter 3 in Gescheider, 1997).

The great cormorant showed the best sensitivity to sound in the region of 1 to 2 kHz, which fits well with the sensitivity range of other diving birds (Crowell et al., 2015). The hearing thresholds from Crowell et al. (2015) are in some cases more than 20 dB higher than the ones derived in this study. This could very well be caused by methodological differences: ABR techniques, like Crowell et al. (2015) used, are well-known for generating higher hearing thresholds than the ones derived using psychophysics (Brittan-Powell et al., 2002, 2005; Crowell, 2016; Wolski et al., 2003). From the study by Crowell et al. (2015), the species that is most phylogenetically similar to the great cormorant is the northern gannet. The great cormorant and the northern gannet may share similarities in adaptations that assist them when they break the water surface (Crowell et al., 2015; Haney and Stone, 1988). It was also found that the gannet might have the ability to control the opening to the ear and prevent water from entering (Crowell et al., 2015), an ability that may be another similarity the great cormorant and the northern gannet share.

The great cormorant is an aquatic bird with in-air hearing abilities very similar to terrestrial species of the same size (Fig. 4). This indicates that in-air hearing is of similar importance for aquatic species as for terrestrial birds. Many aquatic birds are highly social during foraging and breeding, and are known to communicate extensively using acoustic signals. Sound may also be important for the bird to orientate itself in the environmental soundscape, as well as for detecting both potential prey and predators. Further studies on the underwater hearing abilities of marine birds would indicate if hearing is also important for these species while diving.

## MATERIALS AND METHODS

The experimental bird was a male 6-year-old wild-caught great cormorant (*Phalacrocorax carbo*) housed in Kerteminde, Denmark, at the Marine Biological Research Center (University of Southern Denmark). The bird was caught when 5 months old. Its history prior to being caught is unknown, but throughout its time at the laboratory it has remained very healthy. In 2012, the male was joined by a female great cormorant fledgling. The birds were kept in an outdoor 3×3×2.5 m (length×width×height) aviary with an adjacent 1×1×2.5 m quarantine and a 3×5.5×3 m pool cage (with a water depth of 1 m). The birds were trained twice a day using operant conditioning with positive reinforcement techniques. Their daily diets ranged from 250-400 g of fish (capelin, *Mallotus villosus*, and sprat, *Sprattus sprattus*), depending on their motivation and weight, as well as the ambient temperature. The birds were caught and kept under Nature Protection Agency Permit SNS-342-00056 and Ministry of Food and Agriculture Permit 2300-50120-00003-09. The experimental work was performed under Permit (2012-DY-2934-00021/BES) from the Danish Animal Experimentation Inspectorate.

### Experimental procedure

**Fig. 5.**
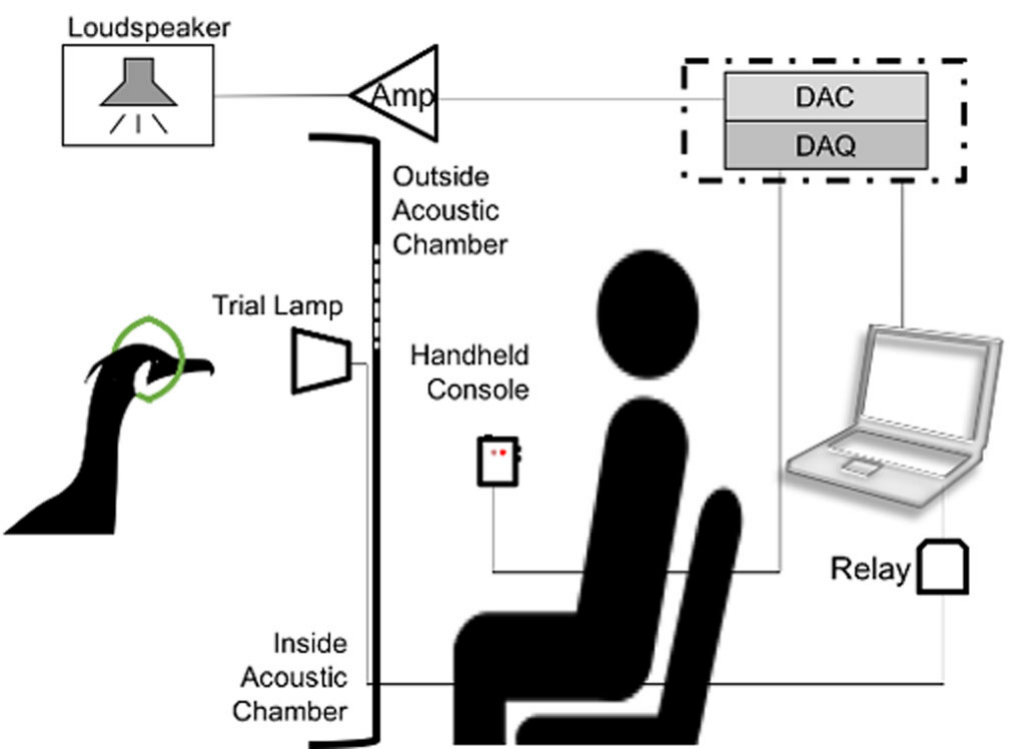
**Experimental setup.** DAQ, data acquisition device; DAC, digital to analog converter; Amp, Amplifier. The dash-dot line around DAQ and DAC represents that both devices were housed in the same setup, but had two different functions.

Each session took place inside a 2.5×1×1 m acoustic chamber connected to the animal's outdoor enclosure. The chamber contained a hoop (13 cm diameter) hanging from the ceiling, with a green lamp 20 cm in front of the hoop and a loudspeaker 20 cm above the hoop (Fig. 5). The experimental system was controlled by a laptop computer running Labview (ver. 2015b; National Instruments, Inc.) for Windows and connected by USB to a National Instruments (NI) DAQPAD 6251. This unit contained both digital inputs and analog outputs. One of the analog outputs was connected via an amplifier to the loudspeaker. Five of the digital inputs were connected to a hand-made console with three buttons and two LED lamps. In addtion, a NI 9481 electromechanical relay, which was attached to an NI USB-9162 carrier, was also connected to the laptop. This relay switched on or off a 12V battery connected to the lamp inside the acoustic chamber. The lamp was used to indicate to the animal the start and end of the the listening period (the time in which a tone was either present or not). A custom-made Labview (ver. 2015b) program was used to control the trials.

Hearing was measured using the methods of constant stimuli (Chapter 3 in Gescheider, 1997). The bird was trained to enter the chamber and to station itself with its head at the centre of hoop. The trainer, visible to the cormorant through a 29×39 cm net panel in the wall 42 cm in front of the bird, would hit a button on a console to initiate a trial. The lamp lit up, indicating to the animal it was time to detect whether a tone was being presented or not. The lamp was automatically switched off after 3 s. It usually took the animal 1-2 s to give a correct response when the signal was present and audible. In GO trials, where a tone-stimulus was presented, the bird was trained to leave the hoop and tap a response target with its beak, which was a red cylinder, 5 cm wide on the bottom, 3.5 cm wide on the top, and 7 cm long, hanging from the ceiling 10 cm to the side of the hoop. In the NOGO trials, when no stimulus was presented, the bird should remain stationary in the hoop for 2 s after the lamp has switched off. For a correct response (denoted as a HIT for GO trials or a correct rejection, or C, for NOGO trials) a broad band (0.5-5 kHz) signal of 0.3 s duration was emitted from the loudspeaker to mark the correct response, followed by the bird being rewarded with a piece of fish. When incorrect responses occurred (denoted MISS for GO trials and FA for NOGO trials), the trainer would wait 3 s before asking the bird to re-station and initiate the next trial. The response of the bird was noted by pressing either the ‘correct’ or ‘incorrect’ buttons on the console.

**Fig. 6.**
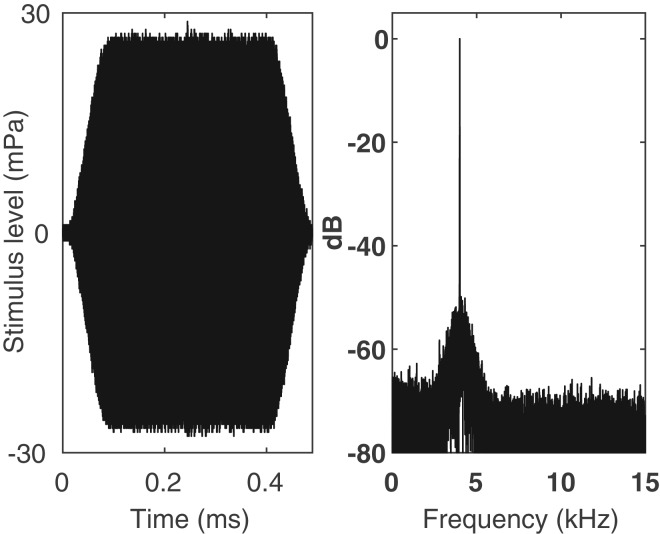
**Sound stimulus.** Recording of sound stimulus at 4 kHz (sampling rate 44.1 kHz, 16 bits). Left: oscillogram. Right: spectrum (FFT size 21712, rectangular window).

The stimulus was a 0.5 s long tone, including 0.1 s long ramp-up and ramp-down segments in the start and end, to avoid spectral smearing (Fig. 6). Test frequencies were: 0.5, 1, 2, 4, and 6 kHz. The order in which the frequencies were tested was randomized. For each frequency, ten sessions were conducted. Each session consisted of five warm-up trials, four cool-down trials and twenty-one data trials. Warm-ups and cool-downs contained signals that were 12 dB higher in intensity than any of the data trials, in addition to a few NOGO trials. These trials were intentionally made easy for the bird to ‘warm up’ before the data trials, and also to give the bird some easy trials at the end (to ‘cool down’). Data trials consisted of 16 GO trials at four intensity levels, spaced by 6 dB, with four trials at each level. These trials were mixed with five NOGO trials. The trial sequence was randomly selected from 12 pre-made trial sequences construced by a Matlab program using Gellerman's (1933) rules for an ‘appropriate’ randomisation of psychophysical trials. The initially completely random schedule was adjusted so that there were never more than three trials in a row of either GO or NOGO trials.

During 54% of the sessions, the trainer wore soundproof headphones and sunglasses to prevent any voluntary or involuntary signalling from the trainer to affect the bird's choice (so-called double-blind procedure; Petrie and Watson, 2013). By using headphones and sunglasses during research sessions, in addition to the pre-made trial sequences, it was possible to eliminate any non-acoustic cues from the trainer that could inidcate to the bird that a signal present or signal absent trial was about to occur.

### Calibration of stimuli and ambient noise recordings

Calibration recordings of stimuli were obtained at regular intervals by placing the microphone underneath the loudspeaker and in the centre of the stationing hoop, at the location where the bird's head would be during each trial. The calibration setup consisted of a PCM recorder (Olympus LS-100 Multi-track Linear Recorder, 16 bits, 48 kHz sampling rate, Line In and recording level 10), a G.R.A.S. 12AA amplifier and a half-inch G.R.A.S., Type 26 AC microphone with a G.R.A.S. AA0009 preamplifier. For the ambient noise and signal calibration recordings, the amplifier was set to +20 dB. Prior to measurements, the system was calibrated by recording the signal from a B&K 4230 calibrator (1 kHz 94 dB re 20 µPa rms; during these recordings the microphone amplifier was set at 0 dB to avoid clipping).

The same recording system was used to measure the ambient noise inside the acoustic chamber at regular intervals, either right before or after the research sessions. To ensure that the microphone itself was not creating too much noise and affecting the noise recordings, a self-noise recording (that is, a recording of the inherent noise produced by the recording system) was made (Fig. 3). This was done by surrounding the microphone with sound insulating material and a piece of PVC tubing, prior to recording inside the sound-proof chamber. The received stimulus level did not vary more than 3 dB around the bird's head for any of the frequencies tested.

### Analysis

Results from each research session were analysed using MATLAB (ver. R2014a; The MathWorks, Inc.). Only sessions with a FA rate (the total number of FAs divided by the total number of NOGOs during a session) of 20-35% were used in the analysis, as an indication that the decision criteria used by the bird was kept relatively constant. The data from all remaining sessions were then pooled for each frequency and the overall HIT rate (the number of HITs divided by the total number of GO trials) was calculated for each intensity level. This data was fitted to a psychometric function using Probit analysis (Finney, 1971). The fit was made with the HIT rates compensated for by the FA rates (Green and Swets, 1966). This resulted in psychometric functions starting out with a HIT rate at the FA rate for low stimulus levels, and approaching a HIT rate of 100% at high stimulus levels (Fig. 1A). The hearing threshold was then defined as the stimulus level where the psychometric function passed the HIT rate at half the interval from the FA rate and 100% HIT rate (*sensu* May et al., 1995).

In addition to this classical psychophysical analysis approach, we used signal detection theory to obtain an unbiased measure of the bird's hearing abilities (*sensu* Chapter 3 in Green and Swets, 1966). First, the FA and HIT rate for a certain frequency and stimulus level were used to estimate the detectability, d’ (Macmillan and Creelman, 2004):

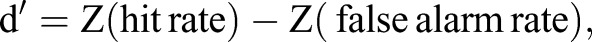
where Z is the inverse of the cumulative normal distribution function (with mean 0 and variance 1; see Chapter 5 in Gescheider, 1997, for details). Once d’ had been calculated, the ROC curve could be estimated as:

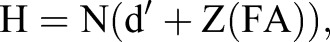
where H is the HIT rate, N is the cumulative normal distribution function with mean 0 and variance 1, and FA is the false alarm rate (Chapter 5 in Gescheider, 1997).

By fitting a linear regression line through the d’ data as a function of signal level for each frequency, we determined the signal level where the regression line equalled 1. This was used as an alternative measure of the hearing threshold for each frequency, which was then compared to the thesholds derived above using classical psychophysics (defined by the stimulus level resulting in a HIT rate right between the FA rate and 100% HIT rate with the psychometric function).

Okanoya and Dooling (1987) determined that for all birds measured so far, excluding the budgerigar (*Melopsittacus undulates*), critical ratios (CRs) follow the same linear trend and can be calculated using:

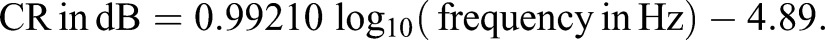
Using this formula, it is possible to estimate the critical ratios for the frequencies tested in this study and thereby determine if the stimulus was masked by background noise during some of the trials (Table 1).

Analysis of acoustic signal and noise levels were made using Matlab (ver. R2014a; The Mathworks, Inc.). Average ambient noise levels were estimated using Welch's method (Proakis and Manolakis, 2006) with an FFT size of 2048, 50% overlap, and a Hann window.

### Statistics

For exploring the influencing factors of HIT rate, we used repeated measures ANOVA with HIT rate as the dependent variable and stimulus level, frequency, false alarm rate, and double blind treatment as explanatory variables. One-way ANOVA (Zar, 1999) was used to test for differences between false alarm rates of non-double blind series and double blind series. All statistical analyses were performed in R (R Core Team, 2014).

## References

[BIO023879C1] Brittan-Powell E. F., Dooling R. J. and Gleich O. (2002). Auditory brainstem responses in adult budgerigars (*Melopsittacus undulatus*). *J. Acoust. Soc. Am.* 112, 999-1008. 10.1121/1.1494807 12243189

[BIO023879C2] Brittan-Powell E. F., Lohr B., Hahn D. C. and Dooling R. J. (2005). Auditory brainstem responses in the Eastern Screech Owl: an estimate of auditory thresholds. *J. Acoust. Soc. Am.* 118, 314-321. 10.1121/1.1928767 16119351

[BIO023879C3] Crowell S. C. (2016). Measuring In-Air and Underwater Hearing in Seabirds. In *The Effects of Noise on Aquatic Life II* (ed. Popper N. A. and Hawkins A.), pp. 1155-1160. New York, NY: Springer New York. 10.1007/978-1-4939-2981-8_144 26611081

[BIO023879C4] Crowell S. C., Wells-Berlin A. M., Carr C. E., Olsen G. H., Therrien R. E., Yannuzzi S. E. and Ketten D. R. (2015). A comparison of auditory brainstem responses across diving bird species. *J. Comp. Physiol. A* 201, 803-815. 10.1007/s00359-015-1024-5 PMC4512887 26156644

[BIO023879C5] Dooling R. J. (1992). Hearing in birds. In *The Evolutionary Biology of Hearing* (ed. Webster D. B., Popper A. N. and Fay R. R.), pp. 545-559. New York, NY: Springer New York.

[BIO023879C6] Dooling R. J. (2002). *Avian Hearing and the Avoidance of Wind Turbines Avian Hearing and the Avoidance of Wind Turbines*. University of Maryland College Park, MD: National Renewable Energy Laboratory.

[BIO023879C7] Dooling R. J. and Okanoya K. (1995). The method of constant stimuli in testing auditory sensitivity in small birds. In *Methods in Comparative Psychoacoustics* (ed. Klump G. M., Dooling R. J., Fay R. R. and Stebbins W. C.), pp. 161-169, New York: Springer-Verlag.

[BIO023879C8] Dooling R. J., Lohr B. Dent M. L. (2000). Hearing in birds and reptiles. In *Comparative hearing: Birds and reptiles*, (ed. Dooling R.J., Fay R.R. and Popper A.N.), pp. 308-359. New York, NY: Springer New York.

[BIO023879C9] Dooling R. J. and Therrien S. C. (2012). Hearing in birds: what changes from air to water. In *The Effects of Noise on Aquatic Life* (ed. Popper A.N. and Hawkins A.), pp. 77-82, New York, NY: Springer New York. 10.1007/978-1-4419-7311-5_17 22278454

[BIO023879C10] Fay R. R. (1988). *Hearing in Vertebrates: a Psychophysics Databook*. Winnetka, IL: Hill-Fay Associates.

[BIO023879C11] Finney D. J. (1971). *Probit Analysis*, 3rd edn Cambridge University Press: Cambridge, UK.

[BIO023879C12] Gellerman L. W. (1933). Chance orders of alternating stimuli in visual discrimination experiments. *J. Gen. Psychol.* 42, 206-208. 10.1080/08856559.1933.10534237

[BIO023879C13] Gescheider G. A. (1997). *Psychophysics, The Fundamentals*, 3rd edn New Jersey: Lawrence Erlbaum Associates.

[BIO023879C14] Green D. M. and Swets J. A. (1966). *Signal Detection Theory and Psychophysics*. New York: Wiley.

[BIO023879C15] Grémillet D., Argentin G., Schulte B. and Culik B. M. (1998). Flexible foraging techniques in breeding Cormorants *Phalacrocorax carbo* and Shags *Phalacrocorax aristotelis*: benthic or pelagic feeding? *Ibis* 140, 113-119. 10.1111/j.1474-919X.1998.tb04547.x

[BIO023879C16] Hall J. W. (2007). *New Handbook of Auditory Evoked Responses*. Boston: Pearson.

[BIO023879C17] Haney J. C. and Stone A. E. (1988). Seabird foraging tactics and water clarity: are plunge divers really in the clear? *Mar. Ecol. Prog. Ser.* 49, 1-9. 10.3354/meps049001

[BIO023879C18] Jackson C. E. (2010). Fishing with cormorants. *Arch. Nat. Hist.* 24, 189-211. 10.3366/anh.1997.24.2.189

[BIO023879C19] Johansen S., Larsen O. N., Dalsgaard J. C., Seidelin L., Boström M., Lunneryd S.-G. and Wahlberg M. (2016). In-air and underwater hearing in the great cormorant (*Phalacrocorax carbo*)*.* *The Effects of Noise on Aquatic life II* (ed. Popper N. A. and Hawkins A.), pp. 505-512, New York, NY: Springer New York. 10.1007/978-1-4939-2981-8_61 26610998

[BIO023879C20] Klink K. B. and Klump G. M. (2004). Duration discrimination in the mous (Mus musculus). *J. Comp. Physiol. A* 190, 1039-1046. 10.1007/s00359-004-0561-0 15480703

[BIO023879C21] Konishi M. (1969). Hearing, single-unit analysis, and vocalizations in songbirds. *Science* 166, 1178-1181. 10.1126/science.166.3909.1178 17775580

[BIO023879C22] Laidre M. E. (2012). Principles of animal communication. *Anim. Behav.* 83, 865-866. 10.1016/j.anbehav.2011.12.014

[BIO023879C23] Langemann U. and Klump G. M. (2001). Signal detection in amplitude-modulated maskers. I. Behavioural auditory thresholds in a songbird. *Eur. J. Neurosci.* 13, 1025-1032. 10.1046/j.0953-816x.2001.01464.x 11264676

[BIO023879C24] Levitt H. (1971). Transformed up-down methods in psychoacoustics. *J. Acoust. Soc. Am.* 49, 467-477. 10.1121/1.1912375 5541744

[BIO023879C25] Macmillan N. A. and Creelman C. D. (2004). *Detection Theory: A User's Guide*, 2nd Edition. New York, NY: Cambridge University Press.

[BIO023879C26] Martin G. R. (1998). Eye structure and amphibious foraging in albatrosses. *Proc. Roy. Soc. B Biol. Sci.* 265, 665-671. 10.1098/rspb.1998.0345

[BIO023879C27] May B. J., Huang A. Y., Aleszczyk C. M. and Hienz R. D. (1995). Design and conduct of sensory experiments for domestic cats. In *Methods in Comparative Psychoacoustics* (ed. Klump G. M., Dooling R. J., Fay R. R. and Stebbins W. C.), pp. 95-108. Basel: Birkhäuser Verlag.

[BIO023879C28] Maiorana V. A. and Schleidt W. M. (1972). The auditory sensitivity of the turkey. *J. Aud. Res.* 12, 203-207.

[BIO023879C29] Moore B. C. J. (2012). *An Introduction to the Psychology of Hearing* 6th Edn Bingley, UK: Emerald Group Publishing Ltd.

[BIO023879C30] Okanoya K. and Dooling R. J. (1987). Hearing in passerine and psittacine birds: a comparative study of absolute and masked auditory thresholds. *J. Comp. Psyc.* 101, 7-15. 10.1037/0735-7036.101.1.7 3568610

[BIO023879C31] Petrie A. and Watson P. (2013). *Statistics for Veterinary and Animal Science*. West Sussex, UK: John Wiley & Sons.

[BIO023879C32] Proakis J. G. and Manolakis D. G. (2006). *Digital Signal Processing*, 4th Edn Pearson, NY.

[BIO023879C33] R Core Team (2014). *R: A Language and ENVIRONment for Statistical Computing*. Vienna, Austria: R Foundation for Statistical Computing http://www.R-project.org/.

[BIO023879C34] Schusterman R. J. (1974). Low false-alarm rates in signal detection by marine mammals. *J. Acoust. Soc. Am.* 55, 845-848. 10.1121/1.1914610 4833081

[BIO023879C35] Trainer J. E. (1946). The auditory acuity of certain birds. *Unpublished doctoral dissertation*, Cornell University (data in Fay, 1988).

[BIO023879C36] Tyack P. (1998). Acoustic communication under the sea. In *Animal Acoustic Communication: Recent Technical Advances* (ed. Hopp S. L., Owren M. J. and Evans C. S.), pp. 163-220, Heidelberg: Springer-Verlag.

[BIO023879C37] Wolski L. F., Anderson R. C., Bowles A. E. and Yochem P. K. (2003). Measuring hearing in the harbor seal (*Phoca vitulina*): Comparison of behavioral and auditory brainstem response techniques. *J. Acoust. Soc. Am.* 113, 629-637. 10.1121/1.1527961 12558298

[BIO023879C38] Zar J. (1999). *Biostatistical Analysis*. NY: Prentice-Hall.

